# Centimeter-Level Orbit Determination for TG02 Spacelab Using Onboard GNSS Data

**DOI:** 10.3390/s18082671

**Published:** 2018-08-14

**Authors:** Kai Li, Xuhua Zhou, Wenbin Wang, Yang Gao, Gang Zhao, Enzhe Tao, Kexin Xu

**Affiliations:** 1 Shanghai Astronomical Observatory, Chinese Academy of Science, 80 Nandan Road, Shanghai 200030, China; xhzhou@shao.ac.cn (X.Z.); zhaogang@shao.ac.cn (G.Z.); tez@shao.ac.cn (E.T.); xkx@shao.ac.cn (K.X.); 2 University of Chinese Academy of Sciences, 19 Yuquan Road, Beijing 100049, China; 3 Key Laboratory of Space Utilization, Technology and Engineering Center for Space Utilization, Chinese Academy of Sciences, 9 Dengzhuang South Road, Beijing 100049, China; wangwenbin@csu.ac.cn (W.W.); gaoyang@csu.ac.cn (Y.G.)

**Keywords:** TG02, multipath, orbit accuracy, POD, centimeter-level

## Abstract

Tiangong-2, the second Chinese manned spacecraft, was launched into low Earth orbit on 15 September 2016. The dual-frequency geodetic GNSS receiver equipped on it is supporting a number of scientific experiments in orbit. This paper uses the onboard GNSS data from 3–31 December 2016 (in the attitude mode of three-axis Earth-pointing stabilization) to analyze the data quantity, as well as the code multipath error. Then, the dynamic and reduced-dynamic methods are adopted to perform the post Precise Orbit Determination (POD) based on the carrier phase measurements, respectively. After that, the orbit accuracy is evaluated using a number of tests, which include the analysis of observation residuals, Overlapping Orbit Differences (OODs), orbit comparison between dynamic and reduced-dynamic and Satellite Laser Ranging (SLR) validation. The results show that: (1) the average Root Mean Square (RMS) of the on-board GNSS phase fitting residuals is 8.8 mm; (2) regarding the OODs determined by the reduced-dynamic method, the average RMS in radial (R), along-track (T) and cross-track (N) directions is 0.43 cm, 1.34 cm and 0.39 cm, respectively, and there are no obvious system errors; (3) the orbit accuracy of TG02 determined by the reduced-dynamic method is comparable to that of the dynamic method, and the average RMS of their differences in R, T, N and 3D directions is 3.05 cm, 3.60 cm, 2.52 cm and 5.40 cm, respectively; (4) SLR data are used to validate the reduced-dynamic orbits, and the average RMS along the station-satellite direction is 1.94 cm. It can be seen that both of these two methods can meet the demands of 3D centimeter-level orbit determination for TG02.

## 1. Introduction

**Figure 1:**
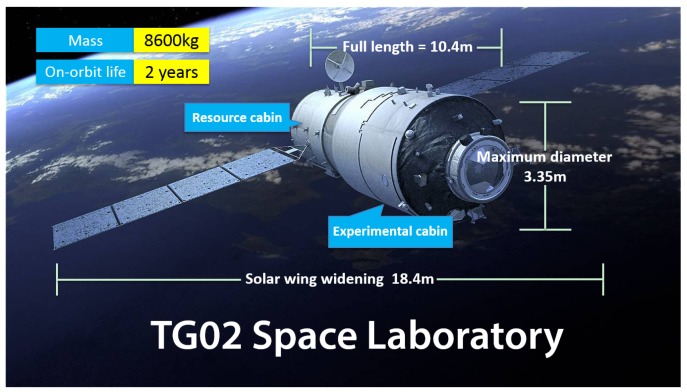
TG02 Spacelab and its main parameters.

Tiangong-2 (TG02) developed by China serves as a truly useful and important spacelab follow-on to Tiangong-1 (TG01). It was launched into near-polar orbits with an initial altitude of about 393 km [1] at the Jiuquan Satellite Launch Centre (JSLC) on 15 September 2016. Its scientific targets are not only to further validate the Rendezvous and Docking (RVD) technology, but also to take on the scientific research and application experiments in the fields of Earth observation from space, space science and technology and aerospace medicine, such as the release of a companion satellite or a cargo spacecraft and its docking. TG02 is similar to TG01 launched in 2011, both are characterized by low orbit and large volume, and all have an experiment cabin, as well as a resource cabin. The main parameters of TG02 are the designed life of two years, the orbital period of 92.2 min, the orbital inclination of 42.8${}^{\circ }$, the mass of 8600 kg, the full length of 10.4 m, the maximum diameter of 3.35 m and the solar wing widening of about 18.4 m; see Figure 1. TG02 is mounted with a dual-frequency geodetic GNSS receiver, Laser Retroreflector Array (LRA), a Laser Lidar Retroreflector Array (LRRA), a cold atomic clock, a spectrograph, a microwave altimeter and other scientific instruments [2].

Like many other Low Earth Orbit satellites (LEOs), the onboard GNSS receiver can provide high-precision and continuous observations from GPS, GLONASS, GALILEO and BEIDOU systems, and the GNSS-based technique is becoming an essential way to perform the Precise Orbit Determination (POD). For this technology, it has been thoroughly and systematically researched [3,4,5,6,7,8], and numerous POD software packages using different algorithms have been developed. Some examples are the Bernese GNSS Software developed at the Astronomical Institute of the University of Bern (AIUB), EPOS developed at GeoForschungsZentrum (GFZ), GIPSY-OASIS developed at the Jet Propulsion Laboratory (JPL), SHORDE-III developed at the Shanghai Astronomical Observatory (SHAO) and PANDA developed at Wuhan University, all of which have been successfully applied to the POD of CHAMP, GRACE, HY2A, ZY3 and many other satellites. Different software packages adopt different force models and data processing strategies. For example, the dynamic POD requires establishing appropriate force models, but generally, the model errors are the major source affecting the orbit accuracy. The kinematic method depends strongly on the uninterrupted geometric information offered by the GNSS data. The POD using the reduced-dynamic method not only employs the advantages of the geometric information, but also gives consideration to the information of the satellite motion; thus, it can improve the orbit quality by adjusting the weight of information derived from the geometric and dynamic information. The outstanding reduced-dynamic method estimates the pseudo-stochastic parameters (e.g., pseudo-stochastic pulses and empirical accelerations) to absorb the force model errors.

Due to the low altitude and large volume of TG02, atmospheric drag perturbation becomes the major perturbative force affecting the accuracy of Orbit Determination (OD), and it is also the acting force that cannot be expressed precisely. It is thus challenging for TG02 to realize the POD with high precision. In this paper, a corresponding software system based on the dynamic and reduced-dynamic methods is developed, then the onboard GNSS data from 3–31 December 2016 are collected to analyze the data quality and perform the POD. At last, the orbit accuracy of TG02 from the internal consistency (fitting of observed data), Overlapping Orbit Differences (OODs), orbit comparison between the dynamic and reduced-dynamic methods and SLR validation is evaluated so that we can demonstrate that the orbit is reliable.

## 2. The Quality of TG02 Onboard GNSS Data

In this section, we first introduce the data source for this study and then assess the quality of TG02 onboard GNSS data mainly in terms of data quantity and multipath errors. The obtained results are then analyzed and discussed.

### 2.1. Data Collection

The TG02 onboard GNSS data (in this paper, we only use the GNSS data acquired from the GPS system for POD) from 3–31 December 2016, were collected from the Technology and Engineering Center for Space Utilization (CSU), Chinese Academy of Sciences (CAS). These data are recorded in the RINEX 2.10 files with a 1-s sampling rate. There are several kinds of observations such as: (1) L1 carrier phase; (2) L2 carrier phase; (3) L1 C/A code; (4) L2 P code; (5) L1 signal amplitude; (6) L2 signal amplitude; (7) Doppler frequency on L1 and (8) Doppler frequency on L2.

**Figure 2:**
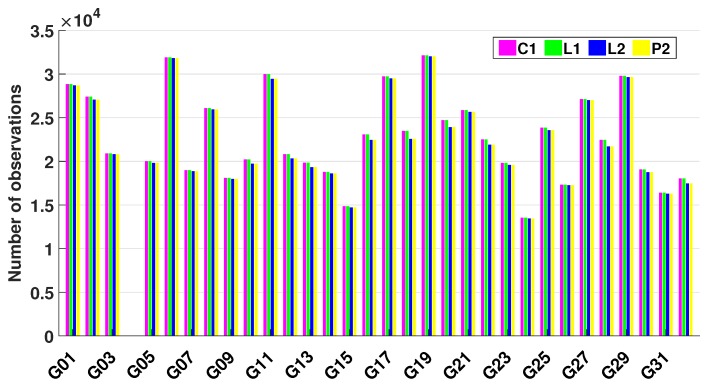
Observations collected by the TG02 onboard receiver for DOY 339, 2016. C/A (purple) and P2 (yellow) code measurements, as well as L1 (green) and L2 (blue) carrier phase observations. No data were collected for G04.

Figure 2 shows the number of GNSS code and phase measurements on Day Of Year (DOY) 339, 2016. It can be seen that the number of these four types of observations (C1/P2/L1/L2) is almost the same.

**Figure 3:**
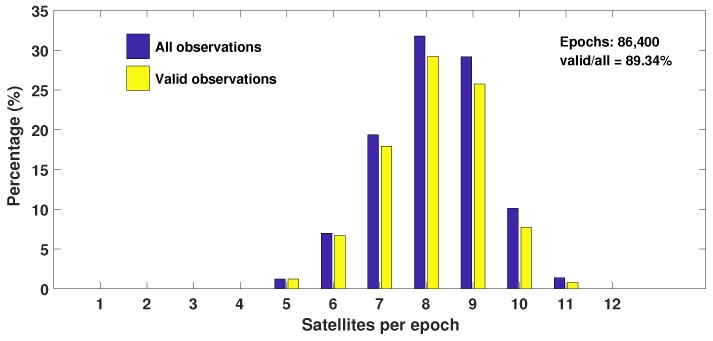
Relationship between the number of observed satellites and the ratio of epochs observed at a certain number of satellites to total epochs for DOY 339, 2016. The blue bar represents the “All observations”, and the yellow bar represents the “Valid observations”.

We know that at one epoch, different numbers of GNSS satellites can be tracked. Figure 3 shows the relationship between the number of observed satellites and the ratio of epochs observed at one certain number of satellites to total epochs for DOY 339, 2016. The label “All observations” refers to the raw data without any processing, and the label “Valid observations” refers to the code and phase measurements with both frequencies. We can see that the minimum number of GNSS satellites is five per epoch, representing the smallest proportion of the total epochs, about 1.24%. The maximum number can reach up to 11. When eight satellites can be tracked, the proportion is the highest, up to 31.8%.

### 2.2. Multipath

**Figure 4:**
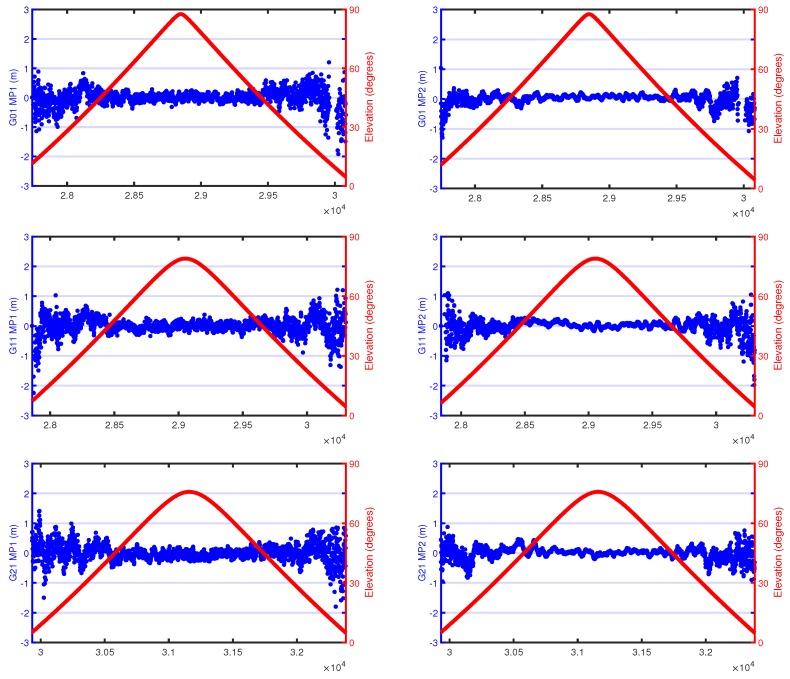
An example of an MPC time series for C1 and P2 observations and elevation-angle variations of complete passes for three different GNSS satellites.

The Multipath Combinations (MPCs) are constructed based on the algorithms used in TEQC [9] using single-frequency code measurements and dual-frequency phase measurements of a continuous ambiguity arc. Constant biases, such as the ambiguities and hardware delay in the satellite and the receiver, are removed by averaging the MPCs of an ambiguity arc. Figure 4 shows an example of MPC time series of C1 and P2 observations and elevation-angle variations of complete passes for three different GNSS satellites with maximum elevation angles close to 90${}^{\circ }$ for DOY 339, 2016. The blue dots represent the MP values, and the dark red lines denote the elevation time series. Due to the rapid movement of TG02, the ascending and descending phases of GNSS satellites are very rapid, and the time interval of a complete satellite pass is only approximately 40 min, which is much smaller than that of ground stations. As illustrated in Figure 4, the lower the elevation angle, the greater the multipath error. Compared with that of C1, MPCs of P2 are smaller for all elevations. The MPCs of C1 and P2 are rather stable, and no gaps are observed at a high elevation.

## 3. Orbit Determination Principle

In this section, we will introduce the methods of dynamic and reduced-dynamic POD. The POD strategy is discussed in the context of the reference frame, force models, observation models and estimated parameters.

### 3.1. Dynamic Orbit Determination

LEOs are always running at high speed on one orbital plane of several hundreds of kilometers away from the Earth’s surface and are affected by many kinds of perturbation forces, mainly including the Earth’s gravity, N-body perturbation, non-spherical perturbation of the Earth, solid tides’ perturbation, ocean tides’ perturbation, atmospheric drag perturbation and solar-radiation pressure perturbation. The equation of motion [7,10] can be described as follows:

(1) $$\ddot{r}=-GM\frac{r}{r^{3}}+f\left(t_{1},r,\dot{r},q_{1},q_{2},\ldots ,q_{d}\right)$$

with initial conditions $r^{\left(k\right)}\left(t_{0}\right)=r^{\left(k\right)}\left(a,e,i,\Omega ,\omega ,M_{0}:t_{0}\right),k=0,1$ (level of time differentiation); where r, $\dot{r}$ and $\ddot{r}$ indicate the position, velocity and acceleration of LEOs; the parameters $a,e,i,\Omega ,\omega ,M_{0}$ are the six Keplerian elements pertaining to epoch $t_{0}$; $q_{1},q_{2},\ldots ,q_{d}$ denote additional dynamical parameters considered as unknowns, e.g., scaling factors of analytically-known accelerations, which describe deterministically the perturbing acceleration acting on the satellite.

The calculation is based on an assumption that the prior orbit $r_{0}\left(t\right)$ is available, e.g., realized by a dynamical fit of LEO positions obtained from a code single-point positioning solution. Therefore, orbit determination discussed in this paper can be considered as an orbit improvement process, i.e., the actual orbit r(t) is expressed as a truncated Taylor series with respect to the unknown orbit parameters $p_{i}$ about the a priori orbit, which is represented by the parameter values $p_{i_{0}}$:

(2) $$r\left(t\right)=r_{0}\left(t\right)+\sum_{i=1}^{n}\frac{\partial r_{0}\left(t\right)}{\partial p_{i}}\left(p_{i}-p_{i_{0}}\right)$$

where n=6+d denotes the total number of unknown orbit parameters, i.e., the six initial conditions for position and velocity and *d* dynamical parameters. The parameters $p_{i}$ include the initial state vector (position and velocity) and all the dynamical parameters $q_{1},q_{2},\ldots ,q_{d}$ that define the satellite force model. The partial derivatives $\frac{\partial r_{0}\left(t\right)}{\partial p_{i}}$ are computed from the so-called variational equations (obtained as the partial derivative of the equation of motion, Equation (1) with respect to the parameters $p_{i}$. The LEO equation of motion, as well as variational equations are usually obtained by a numerical integration.

### 3.2. Reduced-Dynamic Orbit Determination

The state-of-the-art LEO dynamic POD models are hardly known to a level that is comparable to highly accurate tracking data such as high-low GNSS carrier phase measurements or low-low K-band observations. This insufficient knowledge has to be mainly attributed to aerodynamic forces, which are not well predictable at altitudes of 300–600 km due to limitations in the upper atmosphere density models [11] and due to complicated interactions of neutral gases and charged particles with the satellite surface. Therefore, the concept of reduced-dynamic orbit determination [12,13] had been introduced decades ago to exploit the accuracy of the GNSS measurements fully. This has been accomplished by complementing the deterministic model of the spacecraft dynamics by additional stochastic parameters that were adjusted together with the deterministic orbit parameters.

Pseudo-stochastic orbit modeling performed in this paper may be considered as a particular realization of the reduced-dynamic orbit determination technique, which makes use of both the geometric strength of the GNSS observations and the fact that satellite trajectories are particular solutions of a deterministic equation of motion. The instantaneous speed change set in the predefined direction for a certain particular epoch is known as pseudo-stochastic pulses. The pseudo-stochastic pulse parameters are usually set at certain intervals in the R, T and N directions in the satellite co-rotating system [10]. Focusing on one pulse $v_{i}$ at time $t_{i}$ in the predetermined direction e(t), the contribution of $q_{i}=v_{i}$ in f in Equation (1) may formally be written as $v_{i}\delta \left(t-t_{i}\right)e\left(t\right)$, where $\delta (t-t_{i})$ indicates Dirac’s delta distribution, $\delta \left(t-t_{i}\right)=\left\{\begin{array}{cc} 1, & t=t_{i}\\ 0, & t\neq t_{i} \end{array}\right.$. The attribute “stochastic” is chosen because they are characterized both by a priori known statistical properties like an expectation value and an priori weight $\omega_{a_{i}}$ given by an priori variance $\sigma_{a_{i}}^{2}$ with $\omega_{a_{i}}=\frac{\sigma_{0}^{2}}{\sigma_{a_{i}}^{2}}$, where $\sigma_{0}$ denotes the a priori RMS error of unit weight and $\sigma_{a_{i}}$ indicates the RMS error of pseudo-stochastic pulses. The a priori weight $\omega_{a_{i}}$ constrains the estimated parameters on request, preventing them from deviating too much from the expectation value [10]. If $\sigma_{a_{i}}$ is large, the corresponding weight $\omega_{a_{i}}$ becomes small; that is also to say the pseudo-stochastic pulses can absorb the influence of the force model errors, and the role of the dynamical model is weakened. If $\sigma_{a_{i}}$ is small, the corresponding weight $\omega_{a_{i}}$ is large, reveals that the force models are precise and allows only slight changes in velocity. The corresponding variation equation reads as:

(3) $${\ddot{Y}}_{a_{i}}=AY_{a_{i}}+\delta \left(t-t_{i}\right)e\left(t\right)$$

where $Y_{a_{i}}$ may be written as a linear combination of the partial derivatives of the a priori orbit with respect to the six parameters defining the six initial conditions at $t_{0}$. A drawback, however, resides in the fact that $\dot{r}\left(t\right)$ of the improved orbit is discontinuous at the epoch $t_{i}$.

### 3.3. Orbit Determination Strategy

The ionosphere-free combinations of phase and code measurements were taken as the basic observations and then used for the zero-difference (ZD) dynamic and reduced-dynamic OD. The orbit quality depends on the accuracy of force models, the strategies (e.g., parameters estimation) of POD and the quality of GNSS observations applied to both methods [14]. In this paper, some considered force models include gravity field model EIGEN-6S4, solid tides model IERS 2010 conventions, N-body perturbation DE405 and the ocean tides model FES2004. It should be stressed in particular that in the dynamic POD, we use the solar pressure model Box-Wing [15], atmospheric drag model DTM2013 [16] and some empirical forces. However, in the reduced-dynamic POD, solar radiation pressure, atmospheric drag and other unmodeled force errors are replaced by the pseudo-stochastic pulse parameters and empirical accelerations. These parameters are expressed in R, T and N directions with a time interval and a priori Standard deviation (STD). Table 1 shows the strategy for both POD methods.

What needs to be added is that due to the characteristics of the dynamic and reduced-dynamic POD methods, there exist slight differences in the process of estimating parameters. In the dynamic POD, one coefficient of atmospheric drag is estimated every 24 h; constant empirical coefficients in the R and T directions are estimated every 6 h; one solar pressure parameter is estimated every 12 h. In the reduced-dynamic POD, every 6 min, a piece-wise constant acceleration in the R, T and N directions is estimated, and these parameters are used for absorbing the force model errors.

## 4. Results and Analysis

In this section, the dynamic and reduced-dynamic POD are performed. In order to evaluate the orbit accuracy, there are mainly two kinds of means: internal consistency and external consistency. The evaluation of the internal consistency is mainly carried out to analyze the fitting of observation data, such as the statistics of phase residuals or the overlap comparison. The assessment of external consistency is mainly to comparing different orbit products (e.g., obtained from different methods or different agencies), SLR validation. As no reference orbit of TG02 Spacelab has been published yet, this paper will assess the orbit accuracy in terms of the following indices.

### 4.1. Phase Fitting Residuals

**Figure 5:**
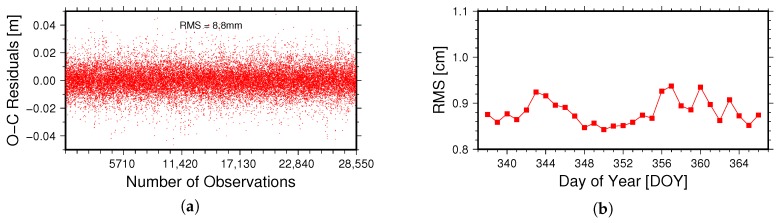
Phase fitting residuals analysis: (**a**) is the post fitting residuals’ distribution diagram on 3 December 2016. (**b**) is the RMS values of phase fitting residuals from 3–31 December 2016.

For POD calculations, most observation errors are corrected during the observation linearization step before normal equation stacking, while the un- or mis-modeled errors, such as observation noise, are still left in the observation equation. From a statistical perspective, part of these errors will be absorbed into the parameters through the estimator, while the rest will still be present in the residuals [8]. However, if we have a good quality of observation data, precise force models and proper parameter estimation, the fitting residuals will be close to the observation noise [3,21]. Usually, large residuals reveal poor POD estimation. However, small residuals can still be obtained even if some errors are not very well modeled, since they can be absorbed into the parameters during the adjustment process. Therefore, residual analysis only can indicate the internal consistency of POD results, but not an external validation. The Figure 5a illustrates the distribution of phase residuals on 3 December 2016, in which the horizontal axis represents the number of observations and the vertical axis represents the phase fitting residuals (in meters). The Figure 5b illustrates the daily RMS of onboard GNSS phase residuals from 3–31 December 2016 (a total of 29 days).

As can be seen from the Figure 5a, the RMS of phase fitting residuals is 8.8 mm on 3 December 2016. It can also be seen from the Figure 5b that the RMS is smaller than 1 cm for each day and the average RMS is 8.8 mm. The internal consistency only reflects the fitting of observation data and cannot evaluate the real accuracy of the satellite orbit quantitatively. Therefore, it cannot serve as the only means to evaluate the orbit quality and must be combined with other evaluation methods to ensure its credibility.

### 4.2. Overlap Comparison

**Figure 6:**
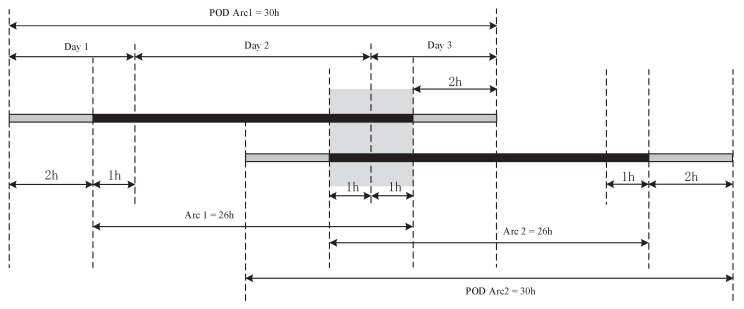
Overlapping arc diagram.

**Figure 7:**
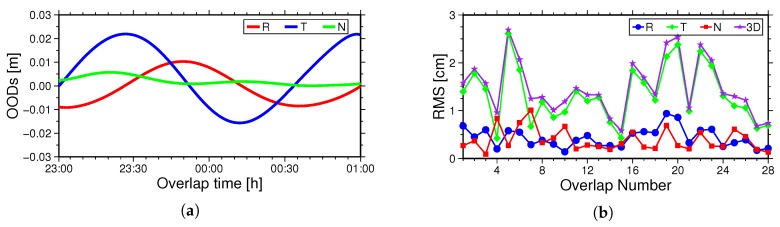
(**a**) is the OODs of the first overlapping arc in the R, T N and 3D directions. (**b**) is the RMS statistics of the total 28 overlapping arcs in the R, T, N and 3D directions.

The orbit overlap comparison is also widely adopted for orbit accuracy validation. In this paper, the arc length is set to 30 h, i.e., from 21:00 of the first day to 3:00 of the third day. In order to avoid boundary effects, only the middle 26-h results are used for the overlap comparison; see Figure 6. The 2-h OODs of two consecutive orbit solutions are used for validation. Although the data from these two hours are the same, the two corresponding orbits are obtained by two independent calculations. The 2-h overlapping orbit can be considered as irrelevant. Therefore, the overlap comparison can preliminarily reflect the orbit determination accuracy [22,23]. For better understanding and expression, we mark the first overlapping arc as 1, and the others can be done in the same manner to the 28th overlapping arc. The Figure 7a indicates the OODs in the R, T and N directions obtained by the reduced-dynamic POD on 3 and 4 December 2016. The Figure 7b indicates the RMS values of 28 overlapping arcs in the R, T, N and 3D directions.

It can be seen from Figure 7 that the OODs between the overlapping Arcs 1 and 2 in the R, T and N directions is 0.68 cm, 1.40 cm and 0.28 cm, respectively. The average RMS of 28 overlapping arcs in the R, T, N and 3D directions is 0.43 cm, 1.34 cm, 0.39 cm and 1.49 cm, respectively. In contrast, the OODs in the T direction are a little large; this may be related to the fact that the forces in the T direction are not well modeled in the reduced-dynamic POD, demonstrating the need for further in-depth research.

### 4.3. Comparison between Reduced-Dynamic Orbit and Dynamic Orbit

**Figure 8:**
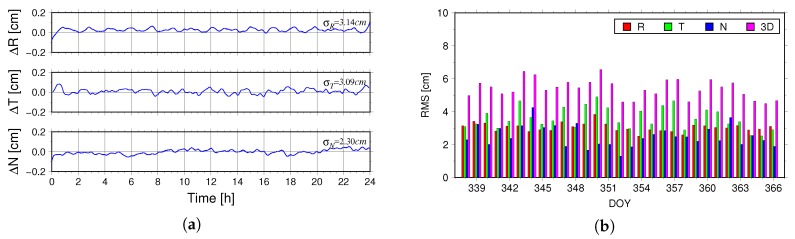
(**a**) is the differences between the dynamic and reduced-dynamic orbit on 3 December 2016. (**b**) is the RMS statistics of the differences between the dynamic and reduced-dynamic orbit for DOY 338–366, 2016.

The reduced-dynamic and dynamic methods are two major algorithms in POD, both of which adopt force models and numerical integration to solve the orbit of LEOs. In contrast to the dynamic POD, the reduced-dynamic method uses less force models and tries to balance the contributions from the force models and the geometric information. Therefore, the orbits obtained by these two methods are more independent. The Figure 8a illustrates the differences between the reduced-dynamic orbit and dynamic orbit in the R, T and N directions on 3 December 2016. The Figure 8b illustrates the RMS values for the differences between the two orbits from 3–31 December 2016.

From the Figure 8a, we can see that on 3 December 2016, the RMS for the differences between the reduced-dynamic orbit and dynamic orbit in the R, T, N and 3D directions is 3.14 cm, 3.09 cm, 2.30 cm and 4.98 cm, respectively. The Figure 8b is the histogram of the differences between the two kinds of orbits from 3–31 December 2016. It can be seen that the average RMS in the R, T, N and 3D directions is 3.05 cm, 3.60 cm, 2.52 cm and 5.40 cm, respectively. Most of the results in the 3D direction are better than 6 cm, which indicates that the two methods can be used to perform the POD for TG02.

### 4.4. SLR Validation

**Figure 9:**
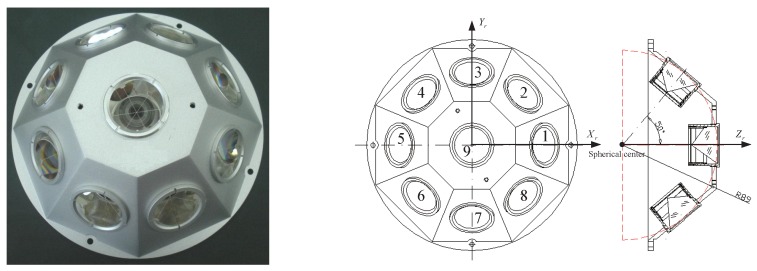
Left is the LRA configuration for TG02. Right is the structural profile of LRA for TG02.

**Figure 10:**
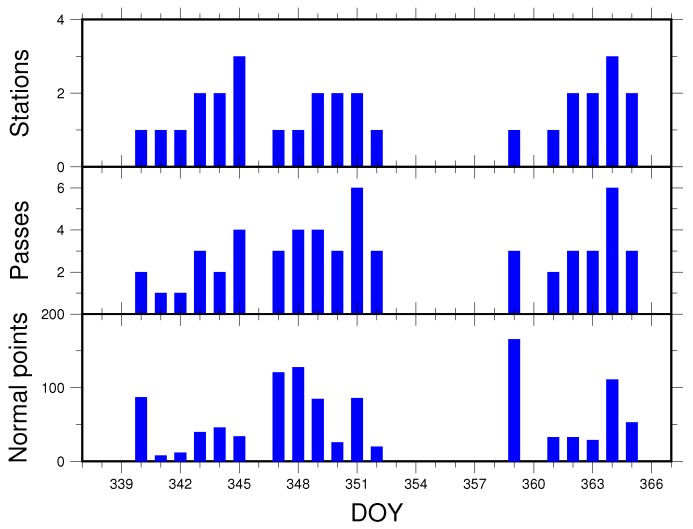
Statistics of the SLR observation data from 3–31 December 2016.

The TG02 Spacelab not only carries the onboard geodetic GNSS receiver, but also the LRA [24]. The LRA developed by SHAO consists of nine cube corner retroreflectors distributed on a hemispherical platform; one is in the middle and the other eight around it; see Figure 9. When TG02 is tracked, the ground stations will receive returns from it, and after pre-processing, we can use the SLR data to carry out the validation apart from the above means. The residuals are the differences between the observed values and the computed values [21,22,25]. From 3–31 December 2016, SHAO organized several SLR stations in China to implement the joint observations for TG02. Affected by the operating status and weather conditions, only Changchun, Shanghai, Kunming and Beijing can provide the SLR data. Figure 10 illustrates the statistics of the SLR data from 3–31 December 2016, including the number of stations, observation passes and normal points for each day.

As can be seen from Figure 10, 1118 normal points and 59 observation passes can be obtained in total from 3–31 December 2016, and there are 12 days without observation records. Table 2 presents an example of SLR residuals corresponding to the reduced-dynamic orbit on 6 December 2016.

**Figure 11:**
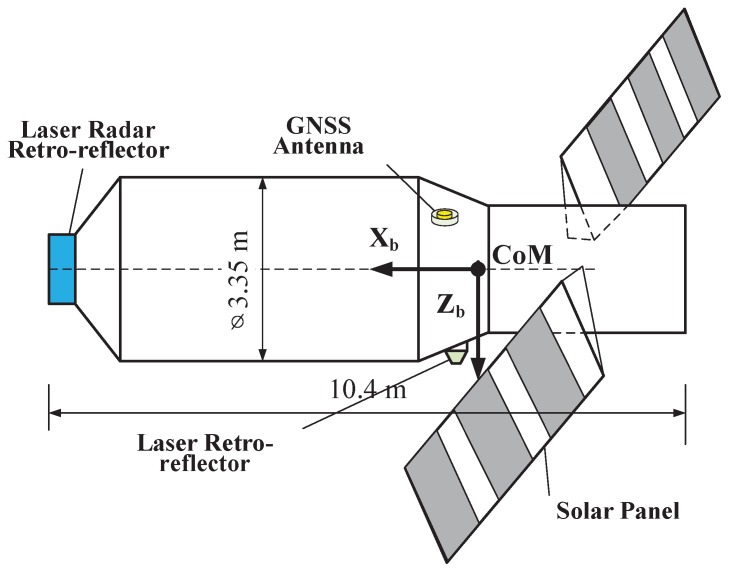
The satellite-body-fixed XYZ coordinates on TG02.

**Figure 12:**
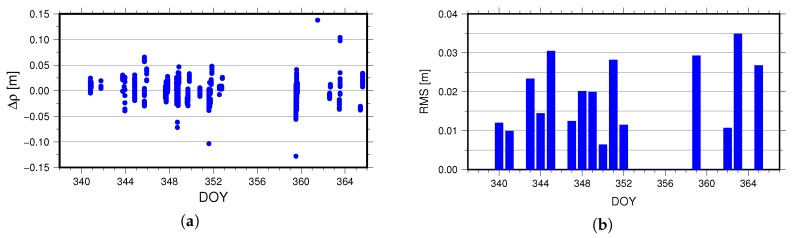
(**a**) is the distribution of SLR residuals for TG02 ZD reduced-dynamic POD during DOY 338–366, 2016. (**b**) is the daily RMS of the SLR residuals for the TG02 ZD reduced-dynamic POD during DOY 338–366, 2016.

Table 2 shows that some residuals are large and reach up to the meter-level. In fact, despite the normal LRRA pointing in the flight direction (see Figure 11), it will also reflect the laser signal from the ground. According to the analysis results, the ground station will receive returns from LRRA and LRA at the same time within the about 2/3 of one flight pass. To implement the POD of TG02, the laser signal from LRA should be retained to produce Consolidated Range Data (CRD) files [26], and the ones from the LRRA should be removed. The Figure 12a illustrates the distribution of the SLR residuals computed by the reduced-dynamic orbit (reject those of larger than 50 cm), and the Figure 12b illustrates the RMS values of the SLR residuals.

As illustrated in Figure 12, the average RMS of SLR residuals for the reduced-dynamic orbit is 1.94 cm. Due to the SLR validation involving a number of corrections, including the influence of the solid tides and ocean tides on the SLR stations, the influence of atmospheric delay on the ranging, the influence of general relativity, the eccentricity correction of the station, the displacement of the station resulting from tectonic plate movement, as well as the existence of observation error in laser ranging, the real value in ranging can be better than 2 cm after correction, so the result of the station-satellite distance validated is reliable.

## 5. Conclusions

In this study, the Chinese TG02 Spacelab onboard GNSS data from 3–31 December 2016 are analyzed. Firstly, the GNSS observation data are evaluated in the context of the data quality, as well as the code measurements multipath errors. Following this, the post POD using dynamic and reduced-dynamic methods is performed based on the carrier phase measurements, then the quality of TG02 ZD POD is assessed by GNSS phase residuals, a comparison between dynamic orbit and reduced-dynamic orbit, an overlap comparison and SLR validation. We draw the following conclusions based on the data analysis and the assessment results.

(1) In terms of the data quality, the number of observed GNSS satellites is from 5–11, and the observation data are continuous. MPC time series are stable, and there are no the elevation-dependent biases in the multipath errors.

(2) Regarding the reduced-dynamic POD, the average RMS for the phase fitting residuals is 8.8 mm; then, a total 28 overlapping arcs obtained from the reduced-dynamic POD are compared, and the average RMS of the differences in the R, T, N and 3D directions is 0.43 cm, 1.34 cm, 0.39 cm and 1.49 cm, respectively. This indicates that the orbit accuracy of TG02 determined by the reduced-dynamic method is reliable.

(3) The onboard GNSS data with a high sampling rate overcome the shortage of the observation data and are conducive to the segmental representation of the dynamical model. Through the comparison between the dynamic and reduced-dynamic POD, the average RMS in the R, T, N and 3D directions is 3.05 cm, 3.60 cm, 2.52 cm and 5.40 cm, respectively. Combined with the characteristics of the reduced-dynamic POD method, the result can well illustrate that the pseudo-stochastic pulses and empirical accelerations set in this method can play a good compensation role. That is to say, using less force models can also achieve high precision orbit.

(4) High-precision SLR observation is an important technical means for validating the satellite orbit accuracy. The reduced dynamic orbit is validated using the SLR data, and the average RMS for the SLR residuals is 1.94 cm. Consequently, the centimeter-level accuracy orbit of TG02, which can be obtained by the reduced-dynamic and dynamic methods, is beneficial to the other scientific missions of TG02.

## Figures and Tables

**Table 1:** GNSS-based TG02 POD strategy.

Model	Description
Reference frame	
Conventional inertial reference frame	J2000.0
Precession and nutation	IERS 2010 conventions [17]
Earth Orientation Parameters (EOP)	IERS C04
Force models	
Mean Earth gravity	EIGEN-6S4 [18]
N-body	JPL DE405 [19]
Relativity	IERS 2010 conventions [17]
Solid tides	IERS 2010 conventions [17]
Ocean tides	FES2004 [20]
Observation models	
Arc length and interval	Reduced-dynamic POD: 30 h, 30 s; dynamic POD: 24 h, 30 s
Onboard GNSS data	Code and phase measurements (C1/P2/L1/L2), 1-s sampling interval, collected from CSU, CAS
GNSS ephemeris	IGS final precise orbit, 15-min sampling interval
GNSS clock	IGS final precise clock, 30-s sampling interval
GNSS satellite antenna PCO/PCV	IGS ATX model igs08.atx
Ionosphere delay	First-order delay eliminated by ionosphere-free linear combination; higher orders are neglected
Estimated parameters	
TG02 initial state	Position and velocity at initial epoch
Receiver clock correction	One per epoch as process noise
Ambiguities	One per satellite pass

**Table 2:** An example of SLR residuals corresponding to the reduced-dynamic orbit on 6 December 2016.

Station Name	Observation Epoch	Residuals (mm)	Station Name	Observation Epoch	Residuals (mm)
7237	63,849	−1126.3	7237	64,029	30.5
7237	63,854	−944.1	7237	75,367	−3865.2
7237	63,869	−0.8	7237	75,370	−3801.7
7237	63,872	−1.3	7821	81,238	−39.5
7237	63,990	21.5	7821	81,249	−35.8
7237	63,994	21.2	7821	81,263	−23.7
7237	64,000	24.8	7821	81,279	−9.6
7237	64,005	22.2	7821	81,293	2.0
7237	64,009	26.5	7821	81,311	17.4
7237	64,015	25.2	7821	81,322	24.5
7237	64,020	28.6	7821	81,336	26.1
7237	64,024	28.6	7821	81,238	−39.5

## Abbreviations

The following abbreviations are used in this manuscript:

TG02	Tiangong-2
POD	Precise Orbit Determination
SLR	Satellite Laser Ranging
RMS	Root Mean Square
R	Radial
T	Along-track
N	Cross-track
JSLC	Jiuquan Satellite Launch Centre
RVD	Rendezvous and Docking
LRA	Laser Retroreflector Array
LRRA	Laser Radar Retroreflector Array
LEOs	Low Earth Orbit satellites
AIUB	Astronomical Institute of the University of Bern
GFZ	GeoForschungsZentrum
SHAO	Shanghai Astronomical Observatory
OD	Orbit Determination
CSU	Center for Space Utilization
CAS	Chinese Academy of Sciences
DOY	Day Of Year
MPCs	MultiPath Combinations
ZD	Zero-Difference
STD	Standard Deviation
EOP	Earth Orientation Parameters
OODs	Overlapping Orbit Differences
CRD	Consolidated Range Data
